# Homotypic dendritic interactions constrain growth and receptor distribution in *Drosophila* T4 neurons without affecting orientation or function

**DOI:** 10.1242/dev.205238

**Published:** 2026-04-08

**Authors:** Melisa Özmen, Nikolas Drummond, Jürgen Haag, Renee Marie Vieria, Jesús Pujol-Martí, Alexander Borst, Tabea Schilling

**Affiliations:** ^1^ Department of ‘Circuits – Computation – Models’, Max Planck Institute of Biological Intelligence, 82152 Martinsried, Germany; ^2^ Graduate School of Systemic Neurosciences, LMU Munich, 82152 Martinsried, Germany

**Keywords:** *Drosophila*, Dendrite development, Homotypic interactions, Motion detection, T4 dendrites

## Abstract

Direction-selective T4 neurons are among the most abundant cells in the *Drosophila* visual system. Although arranged retinotopically, their dendrites do not exhibit classical tiling. Instead, the four T4 subtypes are present once within each of the 750 columns in the fly optic lobe, with their dendrites spanning seven to nine neighboring columns. This results in a dense mesh of overlapping neural processes. Such deviation from classical tiling raises the question of whether homotypic interactions among highly intermingled dendrites such as T4s still contribute to shaping of their dendritic architecture. To address this, we developed Flp2Rescue, a genetic tool that ablates most T4 neurons while stochastically rescuing solitary ones. Our findings reveal that solitary T4 dendrites exhibit significant enlargement, indicating that homotypic interactions normally constrain the dendritic size of T4 neurons. Despite this enlargement, solitary T4 dendrites preserve their main subtype-specific orientations. In the enlarged dendrites we also found a higher number of glutamatergic receptors exhibiting a broader distribution along the dendrite. Surprisingly, these changes do not alter the functional identity of the neurons, meaning solitary T4 neurons continue to respond selectively to motion in their preferred direction.

## Teaser

**Summary:** Use of the genetic tool Flp2Rescue, which aids analysis of solitary neurons, reveals that *Drosophila* T4 dendrites require homotypic neighbors to constrain their dendritic fields, but these interactions are not required for establishing their functional properties.

## INTRODUCTION

Different types of neurons are characterized, amongst others, by their distinct dendrite morphology, which plays an important role in shaping their computation. Therefore, an interesting topic in developmental neuroscience is how dendrites acquire their particular shape. Dendrites serve as the largest membrane compartment in many neurons, extending across vast surface areas interacting with the neighboring neurons as they grow (Grueber et al., 2002; Sugimura et al., 2003; Zhu and Luo, 2004). To ensure proper growth, the molecular interplay between growing dendrites and the extracellular environment must be highly regulated. (Kim et al., 2012; Lefebvre and Marocha, 2020; Sugimura et al., 2007; Gao et al., 2000). Numerous studies have shown that neighboring neurons significantly impact dendritic arbor development across various model organisms. In rats, the orientation of ganglion cell dendrites is strongly influenced by neighboring cells (Perry and Linden, 1982). In goldfish, ganglion cell dendrites expand into areas vacated by ablated neurons (Hitchcock, 1989). A similar effect is also observed in *Drosophila* class IV sensory neurons, which extend their dendrites into regions previously occupied by ablated neighboring cells (Grueber et al., 2003).

One well-characterized outcome of such neighbor-dependent growth is dendritic tiling, whereby dendritic arbors of the same cell type maximize receptive field coverage while minimizing overlap (Grueber and Sagasti, 2010). This is typically achieved through repulsive homotypic interactions**,** where dendrites of the same type actively avoid one another through activity- or contact-dependent mechanisms (Grueber and Sagasti, 2010; Jan and Jan, 2010; Millard and Zipursky, 2008). However, not all neuron types exhibit this avoidance behavior. In some cases, dendrites of the same type can overlap extensively, yet still maintain full coverage of the visual field. The direction-selective T4 neurons of the *Drosophila* visual system represent such an exception. Although T4 neurons are organized retinotopically and their dendritic fields together represent the entire visual field, they do not tile in the classical sense**,** as their arbors intermingle densely with neighboring T4 dendrites**.**

**Fig. 1.**
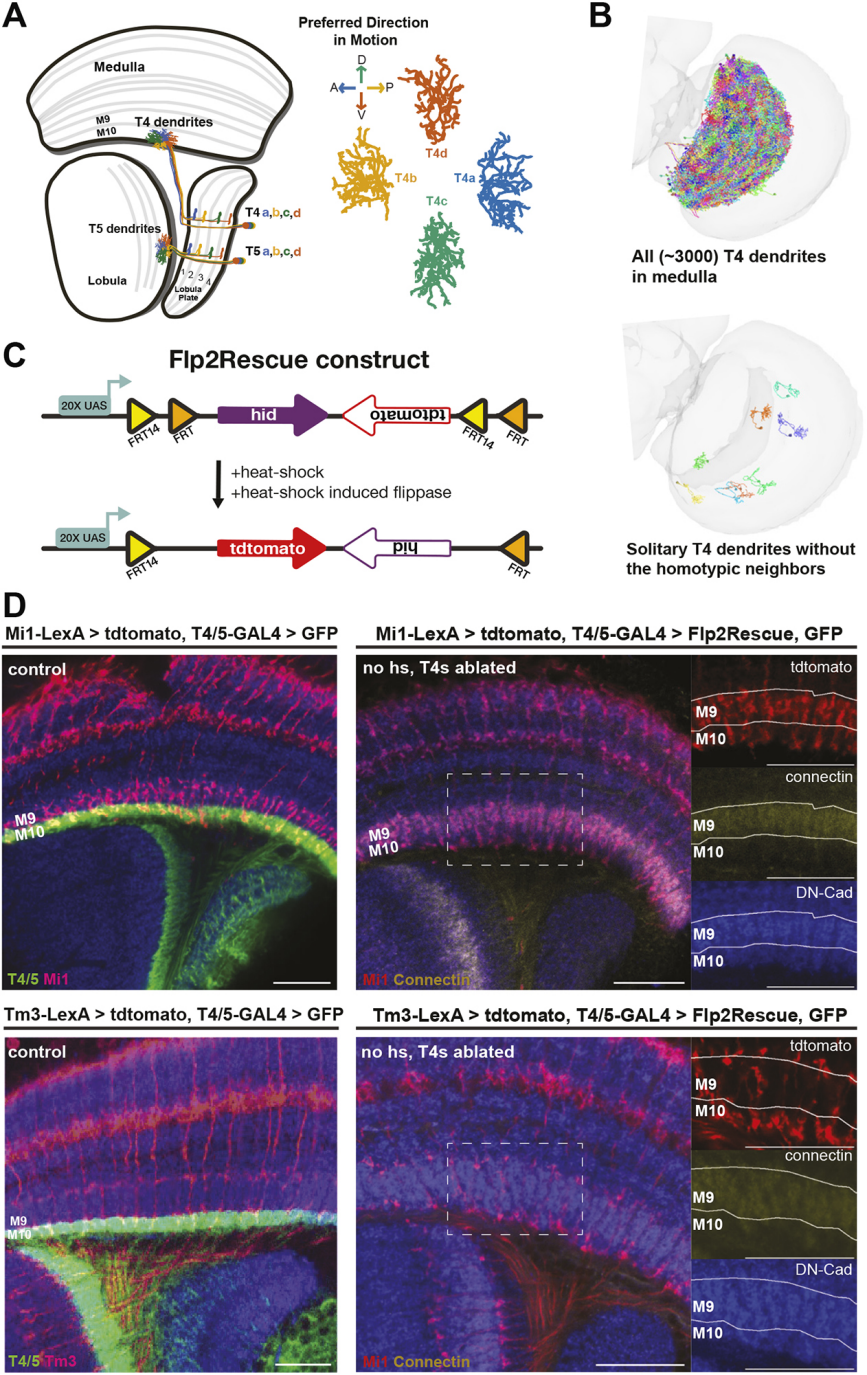
**Flp2Rescue, ablation of T4 neurons and its impact on presynaptic partners.** (A) Schematic of the adult optic lobe (horizontal view) depicting the morphologies of the four T4 subtypes (a, b, c and d). Each of the four lobula plate layers (1-4) receives axons from only one T4 subtype. Arrows indicate the preferred directions of motion for dendrites. A, P, D and V indicates visual field coordinates: anterior, posterior, dorsal and ventral. (B) Adult optic lobe (flat view) (https://flywire.ai) with all T4 dendrites (top) and with solitary surviving T4 dendrites after homotypic neighbor ablation (bottom). (C) Schematic of the Flp2Rescue construct. The pro-apoptotic gene *hid* is positioned under the regulation of the UAS promoter and flanked by half of the flex-switch FRT FRT14. This is followed by CD4::tdTomato in an antisense direction and the other half of the flex-switch FRT FRT14. In the presence of a heat shock-activated flippase, the construct is flipped, which allows CD4::tdTomato expression and *hid* in antisense direction. (D) Adult optic lobe with T4(T5) neurons labeled in green expressing UAS-GFP and ablated expressing UAS-Flp2Rescue. M1 and Tm3 neurons are labeled in red driving the expression of LexAop-tdTomato. Medulla layer 9 is labeled with Connectin. Insets show higher magnification, single-channel images of the boxed area. hs, heat shock. Scale bars: 20 µm**.**

T4 neurons (together with T5 neurons) are the primary direction-selective neurons in the fly visual system. They exist in four subtypes and each subtype is tuned to visual motion in one of the four cardinal directions (left, right, up and down) (Maisak et al., 2013; Pinto-Teixeira et al., 2018; Apitz and Salecker, 2016). T4 dendrites arborize in the most proximal layer of the medulla, responding specifically to moving bright edges (ON pathway). Conversely, T5 dendrites receive their input in the most proximal layer of the lobula, responding to moving dark edges (OFF pathway) (Joesch et al., 2010). Furthermore, the axons of both T4 and T5 neurons exhibit subtype-specific segregation into the lobula plate; subtypes ‘a’ to ‘d’ having their axonal arborizations localized in lobula plate layers 1 to 4, respectively (Fig. 1A) (Fischbach and Dittrich, 1989; Shinomiya et al., 2019; Takemura et al., 2017). In this study, we focus on the T4 dendrites of the ON pathway.

Direction selectivity in T4 neurons arises from the integration of visual information from multiple presynaptic input neurons and these processes have been studied in detail (Ammer et al., 2015; Arenz et al., 2017; Serbe et al., 2016; Takemura et al., 2017a; Lefebvre et al., 2015). T4 dendrites are divided into three functional dendrite compartments (Shinomiya et al., 2019), with all subtypes connecting to their main GABAergic input cells Ct1, Mi4 and C3 at the dendrite base, the main cholinergic input cells Mi1 and Tm3 in the middle part and the glutamatergic input cell Mi9 at the dendrite tips. Furthermore, T4 cells also receive synaptic input from other T4 cells with the same directional selectivity (Shinomiya et al., 2019). Although the four subtypes share a common set of dendritic inputs in the medulla, they respond to different motion stimuli due to their distinct subtype-specific orientations.

T4 neurons are also among the most abundant cell types in the fly optic lobe, again along with T5 neurons. The medulla contains approximately 750 columns, each mirroring an ommatidium in the compound eye, and every column houses one T4 dendrite of each subtype. Because each T4 dendrite spans around seven to nine columns to collect presynaptic input, each column ends up being innervated by roughly 30 densely overlapping T4 dendrites competing for input connections. This dense arrangement contrasts strongly with classical tiling, where neurons of the same type avoid one another to minimize overlap. This raises an intriguing question: in circuits in which dendritic arbors densely overlap, such as in T4 neurons, to what extent do homotypic interactions still contribute to the shaping of dendritic morphology and function? Could interactions among the four T4 dendrites within each column contribute to establishment of their subtype-specific dendritic orientation? Furthermore, does competition between overlapping T4 dendrites for presynaptic input play a role in shaping their functional properties?

To answer these, we developed a genetic tool that ablates all T4 neurons while stochastically rescuing solitary T4s. This allowed us to observe how solitary T4 dendrites develop in the absence of homotypic neighbors (Fig. 1B). We then used confocal and calcium imaging to examine the morphology and the functional properties of these cells. Our results demonstrate that homotypic interactions constrain T4 dendrite fields but these changes surprisingly do not influence their functional properties.

## RESULTS

### Flp2Rescue: a tool to label solitary surviving cells

T4 neurons, together with T5 neurons, are the most abundant neural processes in the *Drosophila* optic lobe, densely populating the medulla, where their growth patterns extensively overlap (Fig. 1A,B). To study how homotypic interactions influence dendritic development, we developed a genetic tool named Flp2Rescue. This tool is based on the FLP/FRT system (Theodosiou and Xu, 1998) and enables cell type-specific ablation while stochastically rescuing a small number of cells. The construct contains a pro-apoptotic gene, *hid* (head involution defective) (Grether et al., 1995), under the control of an upstream activation sequence (UAS) and flanked by two pairs of flippase recognition target (FRT) sites. In addition, it carries a CD4::tdTomato sequence in the antisense direction, allowing surviving cells to be fluorescently labeled following the flipping event (Fig. 1C). To induce recombination at the FRT sites, we used a weak heat shock-inducible flippase under the control of the *hsp70* promoter (hs-FlpG5::PEST). Heat shock-induced expression of flippase mediates site-specific recombination, inverting the construct. Cells in which recombination occurs express tdTomato and survive; those without recombination express *hid* and undergo apoptosis. The number of surviving cells is tightly regulated by the strength and duration of the heat shock as well as the strength of the driver line.

To study the role of homotypic interactions on T4 dendrite development, we aimed to ablate nearly all T4 neurons while rescuing a sparse subset, ideally a few solitary surviving neurons per optic lobe compared to the ∼3000 normally present (Fig. 1B). We focused specifically on T4 dendrites rather than both T4 and T5 because the overall morphology of the optic lobe, particularly the lobula plate, is highly dependent on the presence of these cells (Carrier et al., 2025). We confirmed this by expressing *hid* in T4/T5 cells with the VT043070AD; R39H12DBD Split-Gal4 line, which is active from the late third instar (L3) stage. In these flies, no lobula plate was formed (Fig. S1A). When we drove *hid* expression in T4/T5 cells with an onset of around 18 h after puparium formation (P18) using the VT043070AD; R42F06DBD-Split Gal4 line, lobula plate neuropil was formed in the adult flies, although without clear layer formation (Fig. S1B). This suggests that the early presence of T4/T5 neurons is sufficient to initiate lobula plate formation, even if they are ablated later.

To better interpret the phenotypes of solitary T4 neurons, we first examined whether ablation of T4 neurons affects the layer targeting of their presynaptic inputs. We used the same driver line employed for our ablation experiments (VT016255-AD; R39H12-DBD; ‘early killing’ line), which is described in detail in the following sections. Because the driver line used labels both T4 and T5 neurons, the ablation removes both cell types; however, throughout this section and in the following experiments, we only focus on T4 neurons and therefore will refer to this manipulation as T4 ablation to avoid any confusion. To verify effective ablation, we expressed UAS-GFP under the same T4-Gal4 driver, such that T4 neurons fated to express *hid* were also genetically labeled. In the ablated condition, no GFP signal was detected, confirming successful T4 ablation. We then examined the targeting of T4 input neurons Mi1 and Tm3, expressing tdTomato in the presence and absence of T4 neurons. Neuropil layers were defined using DN-cadherin (Cadherin-N) and Connectin (Gao et al., 2008; Schilling et al., 2019), which strongly labels medulla layer 9 and lobula layer 2. When T4 dendrites were ablated, Mi1 and Tm3 axon terminals still traversed medulla layer 9 and reached medulla layer 10, indicating that their axon targeting is preserved (Fig. 1D). These results demonstrate that ablation of T4 neurons does not disrupt the targeting of their presynaptic inputs to M10. Importantly, this allowed us to exclude altered input layer targeting as a cause of any change observed in solitary T4 neurons in the subsequent experiments.

### T4 dendrites exhibit overgrowth and mistargeting when neighbors are absent in the late developmental stages

**Fig. 2.**
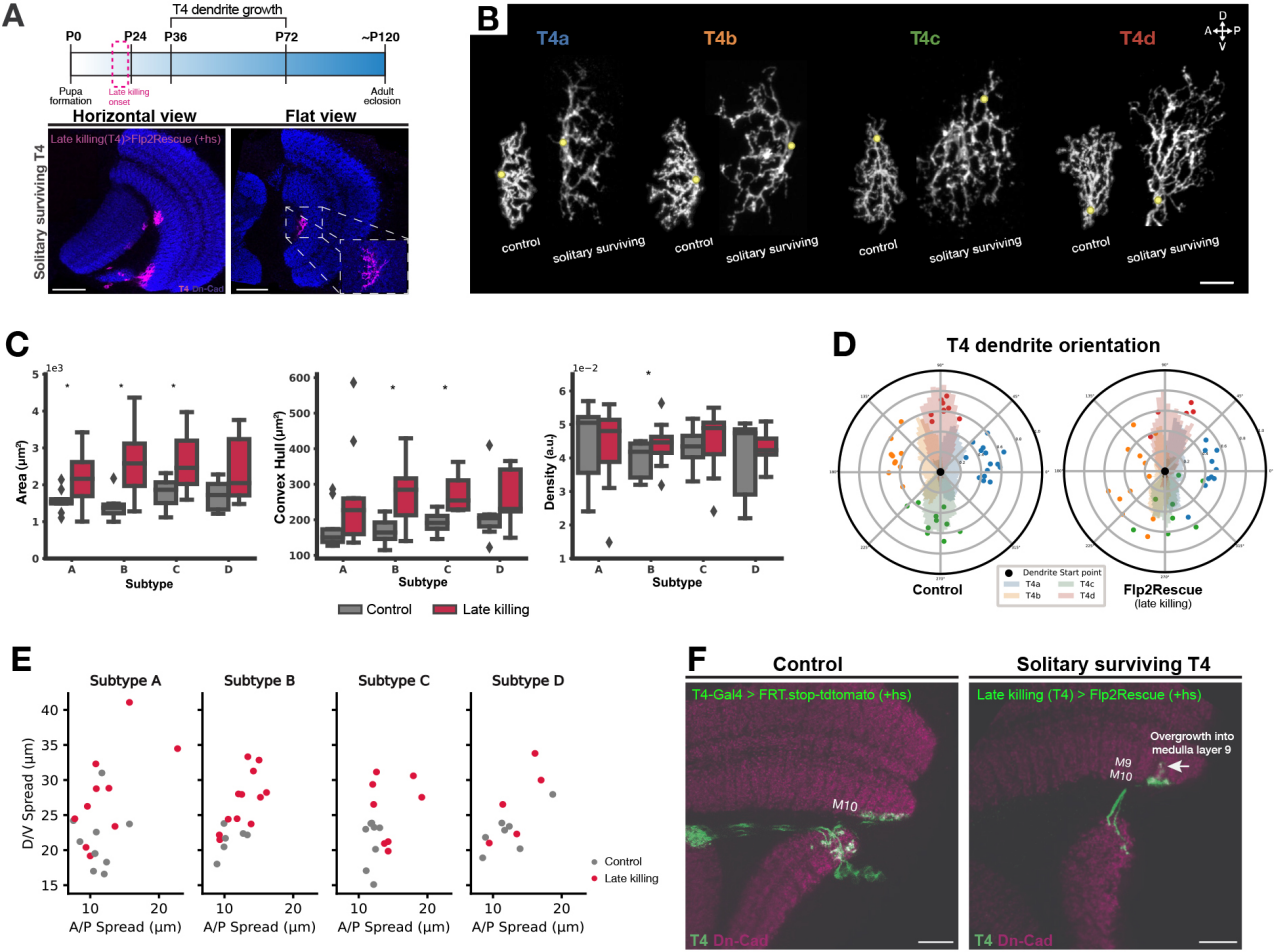
**Morphological changes in solitary surviving T4 dendrites following late ablation of T4 neighbors.** (A) Top: Developmental timeline of T4 neurons with the onset of the late killing driver line. Bottom: Adult optic lobes with solitary surviving T4 dendrites (magenta) from horizontal and flat views. Late killing T4-GAL4>Flp2Rescue (+hs). Scale bars: 20 µm. (B) Comparison of single hs-Flippase; late killing T4-GAL4-driven solitary surviving T4 dendrites with control T4 dendrites. Yellow dots show dendrite entry points. A, anterior; D, dorsal; P, posterior; V, ventral. Scale bar: 5 µm. (C) Quantification of dendritic area (left), volume (middle), and density (right) across all four T4 subtypes (A-D) in control (gray) and late killing (magenta) conditions. Solitary T4 dendrites in the late killing condition show significantly larger dendritic area and volume across all subtypes compared to controls. *P*-values from permutation tests, Holm-corrected across subtypes (A-D). *P≤0.05. Control dendrites: *n*_T4a_=15, *n*_T4b_=10, *n*_T4c_=9, *n*_T4d_=10. Late killing solitary surviving dendrites: *n*_T4a_=14, *n*_T4b_=14, *n*_T4c_=8, *n*_T4d_=6 dendrites. a.u., arbitrary units. Box plots show the median (center line), the interquartile range (box), and whiskers extending to the most extreme data points within 1.5× the interquartile range. Points beyond the whiskers are plotted as outliers. (D) Average dendrite orientation of solitary surviving and control T4 dendrites (late killing). The angle between the start point of the dendrite and each pixel is calculated, relative to the imaging plane. Histogram plots show the angles of pixel for each subtype; angles are binned into 100 bins between 0° and 360°. Dots represent the mean angle of individual neurons. (E) Scatter plots showing the anterior-posterior (A/P) and dorsal-ventral (D/V) dendritic spread in control (gray) and late killing (magenta) conditions for each T4 subtype (A-D). In the late killing condition, solitary surviving T4 dendrites show elongation along both axes compared to controls. Each point represents one dendrite. (F) Horizontal view of adult optic lobe with single T4 dendrites (green). Arrow indicates overgrowth into deeper medulla layers. Scale bars: 20 µm.

T4 cell progenitors emerge during L3. As an initial step, nascent T4 neurons extend their immature dendrites into M10, where they remain in a single column without further growth. Around P36, dendrite outgrowth begins simultaneously across the T4 population and continues until approximately P72 (Hörmann et al., 2020). This is followed by synaptic pruning, which refines T4 dendritic architecture to achieve their mature morphology (Fig. 2A, upper panel). To investigate the role of homotypic interactions in shaping T4 dendrite development, we aimed to grow T4 dendrites in an environment in which neighboring T4 dendrites are ablated. The timing of this ablation is crucial and involves several considerations. First, heat shock to induce flippase-mediated inversion can only be applied from late L3 on, when T4 neurons begin to emerge. Therefore, the Gal4 driver line used for ablation must not be active prior to this stage. Second, to effectively disrupt early homotypic interactions, ablation must occur before the dendritic growth starts.

**Fig. 3.**
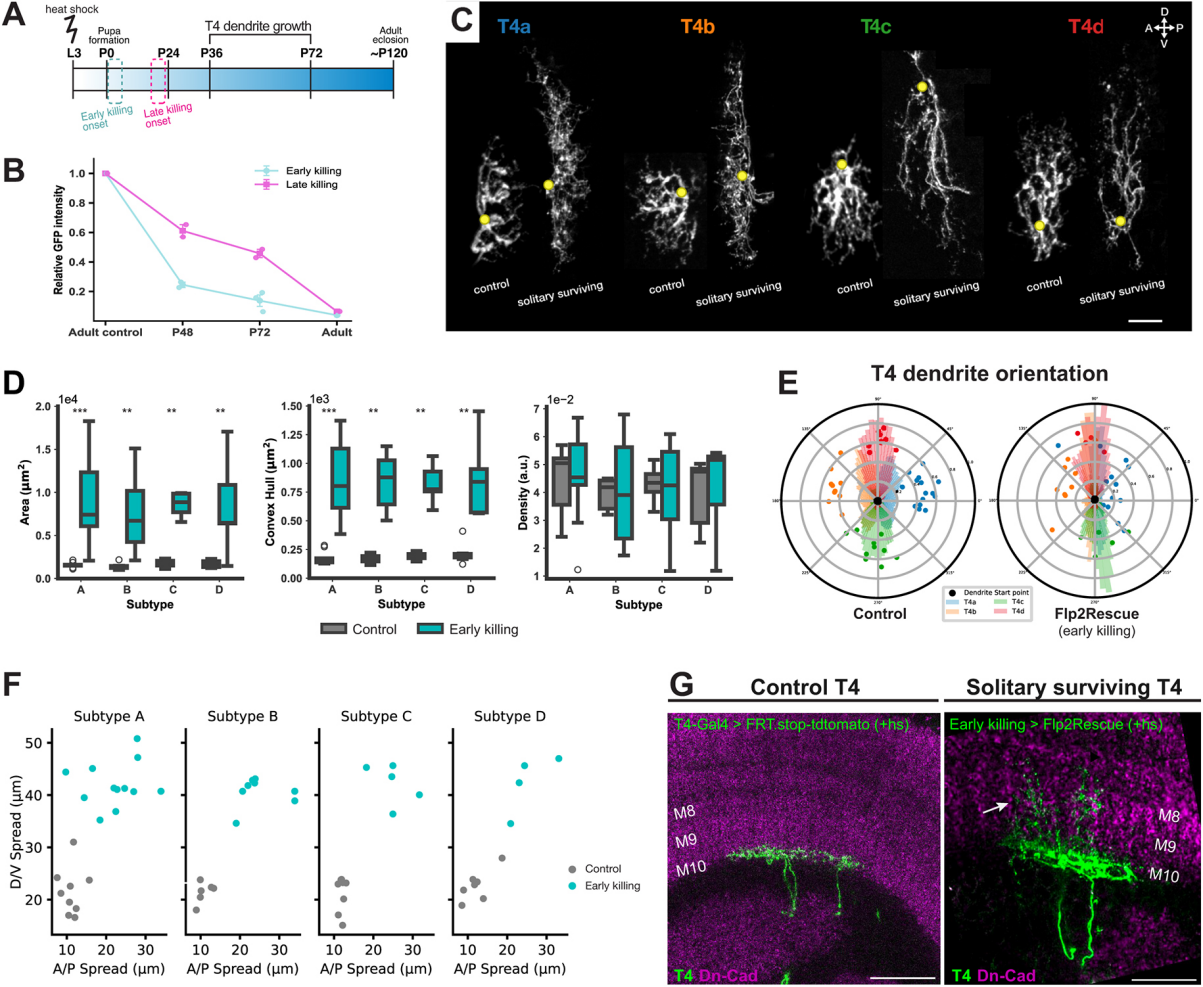
**Morphological changes in solitary surviving T4 dendrites following earlier ablation of T4 neighbors.** (A) Schematic timeline showing the onset windows of the driver lines. Blue and magenta dashed boxes indicate early and late ablation windows, respectively. (B) Relative GFP intensity of T4 dendrites in M10 as a proxy of ablation with late and early killing driver lines at different developmental stages. (C) Comparison of single hs-Flippase; early killing T4-Gal4-driven solitary surviving T4 dendrite skeletons with control T4 dendrites. A, anterior; D, dorsal; P, posterior; V, ventral. (D) Quantification of dendritic area (left), volume (middle) and density (right) across all four T4 subtypes (A-D) in control (gray) and early killing (cyan) conditions. Solitary T4 dendrites in the early killing condition show significantly larger area and volume across all subtypes compared to controls. *P*-values from permutation tests; Holm-corrected across subtypes A-D. **P≤0.01, ***P≤0.001. Control dendrites: *n*_T4a_=15, *n*_T4b_=10, *n*_T4c_=9, *n*_T4d_=10. Early killing solitary surviving dendrites: *n*_T4a_=12, *n*_T4b_=10, *n*_T4c_=6, *n*_T4d_=6 dendrites. a.u., arbitrary units. Box plots show the median (center line), the interquartile range (box), and whiskers extending to the most extreme data points within 1.5× the interquartile range. Points beyond the whiskers are plotted as outliers. (E) Average dendrite orientation of solitary surviving and control T4 dendrites (late killing). The angle between the start point of the dendrite and each pixel is calculated, relative to the imaging plane. Histogram plots show the angles of pixel for each subtype, angles are binned into 100 bins between 0° and 360°. Dots represent the mean angle of individual neurons. (F) Scatter plots showing the anterior-posterior (A/P) and dorsal-ventral (D/V) dendritic spread in control (gray) and early killing (cyan) conditions for each T4 subtype (A-D). In the early killing condition, surviving solitary T4 neurons show greater dendritic expansion along both axes compared to controls. Each point represents one dendrite. (G) Horizontal view of optic lobe with control and early killing driven solitary surviving T4 dendrites. Arrow indicates overgrowth into deeper medulla layers. Scale bars: 20 µm.

Therefore, we used a T4-specific Split-Gal4 line, VT016255AD; VT012314DBD (referred to as ‘late killing’) with an onset around P15-P20 (Fig. S2A). This gave us the time window to ablate T4 neurons prior to the dendritic growth. Importantly, complete ablation of T4 neurons with this line did not result in any disruption in the adult lobula plate. The presence of viable T5 cells was sufficient for the lobula plate to form its characteristic four distinct layers (Fig. S2C). As a control, we used the same driver line driving the expression of stop-tdTomato. We next assessed the apoptosis efficacy and the clearance of dendrite remnants over time following *hid* induction. To directly visualize and quantify ablation, we co-expressed UAS-GFP under the same T4 driver used for ablation, thereby labeling *hid*-expressing (and dying) T4 neurons. We measured GFP signal intensity at multiple developmental stages as a proxy for the amount of remaining T4 dendritic material, averaging measurements across two or three brains per condition. The GFP signal decreased corresponding to ∼54% loss by P72 and ∼95% loss in the adult (Fig. S2C, Fig. 3B). This showed that, although the *hid* expression begins around P15-20, ablation and clearance of dendrite remnants required several additional hours.

To analyze dendrite morphology, we imaged solitary surviving T4 dendrites flat within the upper region of medulla for morphology analysis (Fig. 2A, lower panels). In the absence of homotypic interactions, we observed an increase in the size of the surviving dendrites (Fig. 2B). The dendrites covered larger areas with increased elongation in both anterior-posterior (A-P) and dorsal-ventral (D-V) axes. Dendrite volume was also increased but density remained similar to that of control T4 dendrites indicating that the increased territory is not due to sparse, elongated branches but rather reflects a well-structured, fully arborized expansion. Interestingly, in the absence of homotypic interactions, T4 dendrites on average maintained their main subtype-specific orientation. However, in more than 50% of the horizontal subtypes (subtypes a and b) we observed a growth bias favoring the D-V axis (Fig. 2C-E, Fig. S2D). We next conducted imaging of solitary surviving dendrites from the horizontal view to see whether they exhibited any mistargeting phenotype. We observed that T4 dendritic branches extended laterally into the deeper medulla layers, where T4 cells are not typically found. Some branches targeted medulla layer 9, indicating potential expansion beyond their typical dendritic territories (Fig. 2F). Because this phenotype was observed in a different imaging configuration and dataset from those used for the quantitative analyses, a direct quantification of deeper medulla targeting across solitary surviving dendrites was not feasible. We therefore present this panel as a representative example illustrating the phenotype.

Our results suggest that the primary orientation of T4 dendrites develops independently of homotypic interactions among T4 neighbors. T4 dendrites appear to constrain each other's size and restrict their targeting into deeper medulla layers. At this stage, we cannot entirely rule out the early influence of homotypic interactions on T4 dendrite development. The Gal4 line we used to initiate the *hid* expression in T4 cells started around P20. Transcription and translation of *hid* mRNA into functional protein usually begins within 2 h after Gal4 activation (Milán et al., 1997). Once Hid protein expression reaches a threshold, apoptosis is initiated rapidly, which usually occurs 2-4 h after *hid* expression begins. Observable apoptotic features such as membrane blebbing appear 4-8 h post-induction (Grether et al., 1995; Goyal et al., 2020). Complete clearance of apoptotic cells may take several additional hours, varying with the specific tissue and developmental stage. This explains why around half of the T4 population was still present at P48. Therefore, we cannot rule out potential interactions between T4 cells from L3 to P48, as well as the possible influence of dendrite remnants on the surviving dendrites until their significant removal by P72. To address these uncertainties, we aimed to initiate apoptosis in T4 cells at an earlier developmental stage.

### Early ablation of homotypic interactions enhances overgrowth phenotypes in solitary T4 dendrites

We next combined VT016255-AD and R39H12-DBD to generate a new T4-specific Gal4 line (referred to as ‘early killing’), with an earlier onset. Expression of the early killing driver line started around P0, enabling us to ablate T4 cells approximately 20 h earlier compared to the late killing line (Fig. 3A, Fig. S3A-C). Furthermore, we could still apply the heat shock to late-stage larvae ensuring sufficient time between recombination and subsequent Gal4 expression (Fig. 3A). The driver line showed some expression also in T5 cells, but it was weaker and delayed compared to that in T4 cells, allowing the formation of a properly structured adult lobula plate (Fig. S3C). However, obtaining solitary surviving cells at this stage was challenging, likely due to the short recombination window and the large number of progenitor cells still present at that developmental time point.

As we did for the late killing line, we first assessed the efficiency and timing of *hid* expression under control of the early killing line. We observed that T4 cell density was significantly decreased already by P48 with ∼75% being ablated. The ablation increased to ∼86% by P72 and to ∼97% in the adult (Fig. 3B, Fig. S3B,C). We then located single T4 dendrites to analyze their orientation and morphology when T4 neighbors were ablated earlier (Fig. 3C). Our solitary surviving dendrite analysis showed that earlier ablation of T4 homotypic interactions led to more pronounced alterations on T4 dendrite morphology. We observed extreme increases in both dendrite area and volume with significant elongation along the D-V and A-P axes. Despite these changes, the density of T4 dendrites remained again similar to that of control flies. Furthermore, ablating homotypic interactions earlier still did not disrupt the main subtype-specific orientation of T4 dendrites. However, we observed a more pronounced dorsal-ventral growth across all subtypes (Fig. 3D-F, Fig. S3D). The overgrowth into medulla layer 9 observed with the late killing-driven ablation was significantly more in the case of early ablation. Solitary surviving dendrites showed further elongation of their branches and reached into medulla layer 8 (Fig. 3G). These results suggest that early ablation of neighboring T4 cells leads to a more pronounced dendritic overgrowth in the surviving cells compared to late ablation, indicating that the timing of homotypic interaction loss influences the extent of the effect in the T4 dendrite morphology. These results further demonstrate that T4 dendrites can establish their distinct orientation independently of T4–T4 interactions during the period of dendritic growth.

### Competition for synapse formation among T4 cells refines their functional dendritic compartments

**Fig. 4.**
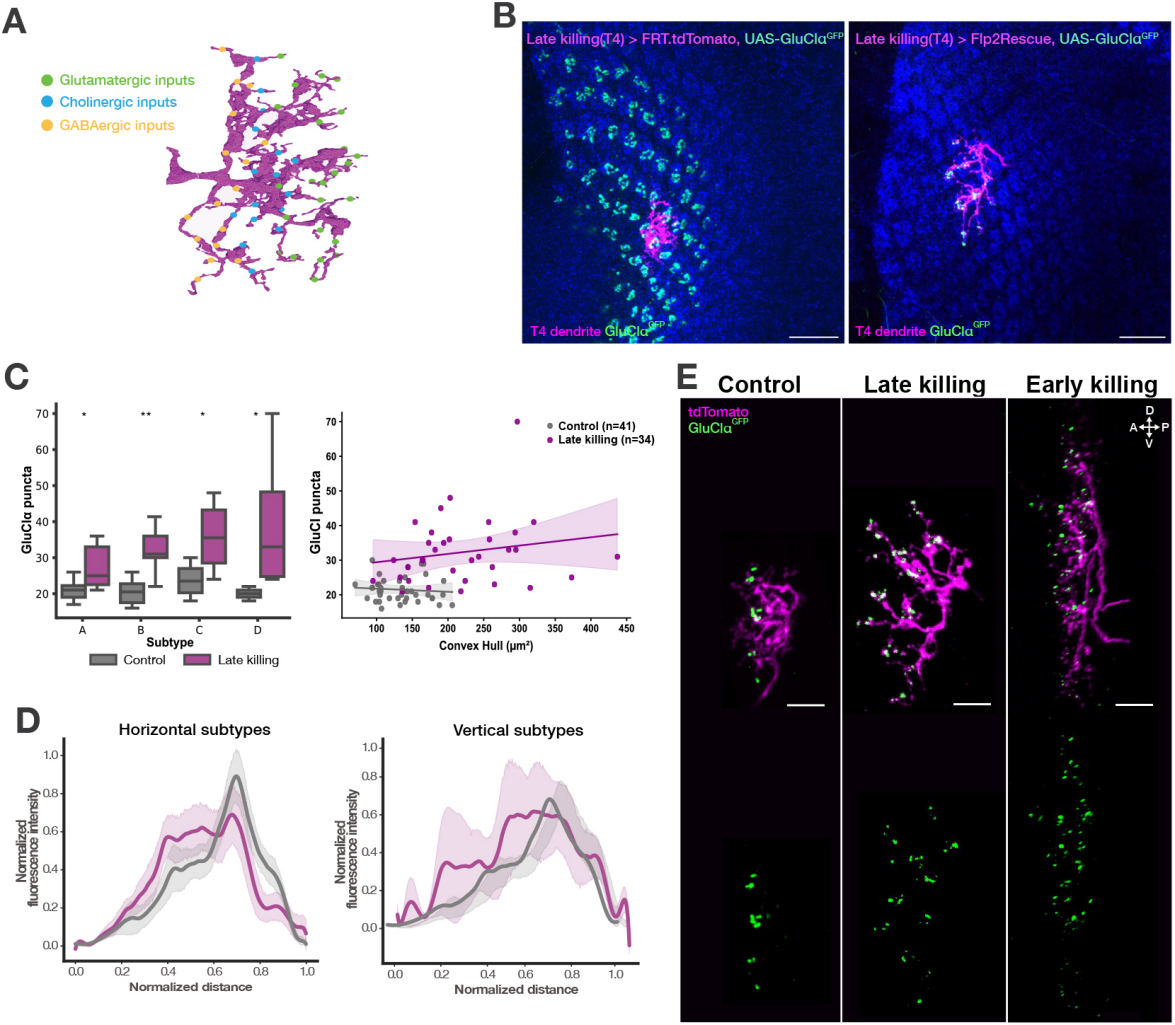
**Glutamatergic synapses after eliminating homotypic interactions.** (A) Electron microscopy reconstruction of a T4a dendrite (https://flywire.ai) with synaptic input distribution. Glutamatergic synapses from Mi9 (green) are located on the distal side, cholinergic synapses from Mi1 and Tm3 (blue) are located at the center, and GABAergic synapses from CT1, Mi4 and C3 (yellow) are at the proximal side of the T4 dendrite. (B) Left: Single T4 dendrite in flat medulla labeled with tdTomato (magenta) with GluClα receptor puncta in all T4 dendrites labeled with GFP (green). Right: Solitary surviving T4 dendrite (late killing) (magenta) with GluClα receptor puncta (green). Scale bars: 10 µm. (C) Left: Comparison of GluClα puncta number on single T4 dendrites between late killing solitary surviving dendrites and control dendrites. Each subtype is compared individually. *P*-values from permutation tests; Holm-corrected across subtypes (A-D). *P≤0.05, **P≤0.01*.* Control dendrites: *n*_T4a_=12, *n*_T4b_=10, *n*_T4c_=10, *n*_T4d_=9. Late killing solitary surviving dendrites: *n*_T4a_=11, *n*_T4b_=13, *n*_T4c_=5, *n*_T4d_=5 dendrites. Box plots show the median (center line), the interquartile range (box), and whiskers extending to the most extreme data points within 1.5× the interquartile range. Right: Correlation between dendrite volume and GluClα puncta number in late killing solitary surviving dendrites and control dendrites. All subtypes plotted together. Dots represent individual dendrites. Solid lines indicate the linear regression fit for each group, and shaded areas represent the 95% confidence interval of the fitted regression line. (D) Quantification of GluClα distribution over the whole dendritic length (normalized distance) averaged across several T4 dendrites from all subtypes. Control dendrites: *n*_horizontal_=12, *n*_vertical_=11. Late killing solitary surviving dendrites: *n*_horizontal_=16, *n*_vertical_=12. All dendrites were aligned pointing to the right with the most proximal point at 0.0 and the most distal point at 1.0. Mean profiles are shown as lines and variability is displayed as a shaded band representing mean±s.e.m. Mann–Whitney test, horizontal subtypes (centroid *P*=0.0007; distal fraction *P*=0.00094); vertical subtypes (centroid *P*=0.095; distal fraction *P*=0.224). (E) Comparison of single control and early and late killing solitary surviving T4b dendrites expressing UAS-GluClα^GFP^. A, anterior; D, dorsal; P, posterior; V, ventral. Scale bars: 5 µm.

All four T4 subtypes receive input from the same set of presynaptic partners but in a distinct spatial order (Borst, 2018; Haag et al., 2016), suggesting potential competition among T4 neurons for synaptic connections. Under normal conditions, each input neuron forms synapses with multiple T4 dendrites, for example typically around four Mi1-to-T4 connections within a single medulla column. At the same time, each T4 dendrite has around three or four Mi1 input partners. In our sparse condition, in which only one T4 neuron survives in the column, this typical connectivity is disrupted. With no neighboring T4 cells present, presynaptic neurons might either re-distribute more synapses onto the single remaining T4 or fail to form proper connections due to a lack of the usual spatial cues. This raises key questions about how synaptic input is reorganized when competition and local targeting mechanisms are altered. To address this, we used the GluClα receptor as a proxy for glutamatergic synapses, as it is the primary receptor for glutamatergic Mi9-to-T4 synapses (Fendl et al., 2020) (Fig. 4A). GluClα is predominantly localized at the tips of T4 dendrites and begins to accumulate on the dendrite in the late pupal stages (Sanflippo et al., 2024). The timing of GluClα accumulation allows neighboring T4 dendrites to be ablated prior to the onset of GluClα expression in the rescued cells. To image GluClα distribution, we co-expressed UAS-GluClα^GFP^ with the Flp2Rescue construct in T4 cells using the late killing driver line. Overexpression of GFP-tagged GluClα labels glutamatergic synapses without altering their density (Fendl et al., 2020). We used two different experimental setups as controls. First, we expressed UAS-GluClα^GFP^ together with a stop-tdTomato cassette, which drives GluClα expression in all T4 dendrites while restricting tdTomato to a sparse subset (Fig. 4B, left). This setup allowed us to observe the GluClα pattern in the M10. However, in this setup, receptor puncta on the control dendrites could not be reliably quantified due to high background signal from surrounding GluClα in unlabeled dendrites. To enable quantification of receptor puncta on individual control dendrites, we introduced Gal80 to restrict Gal4 activity, thereby driving UAS-GluClα:GFP and UAS-tdTomato expression in a sparse population of T4 neurons. This approach allowed for clear visualization and quantification of receptor puncta in isolated dendrites (Fig. 4B, right). In solitary surviving T4 dendrites, GluClα puncta were no longer observed in the surrounding area, confirming the effective ablation of neighboring T4 neurons.

We observed an increase in the number of GluClα puncta in solitary surviving dendrites (Fig. 4C). While this increase could logically result from the larger dendrite size, no clear correlation was found between dendritic size and the puncta number (Fig. 4C). For the spatial distribution of GluClα, we analyzed T4a and T4b dendrites together as horizontal dendrites and T4c and T4d as vertical dendrites, since they mirror each other morphologically and do not differ in synapse number. In solitary surviving dendrites, GluClα puncta showed reduced distal enrichment compared to the control dendrites, significant in horizontal subtypes (*P*=0.00094) but not in vertical subtypes (distal fraction *P*=0.224) (Fig. 4D). In the absence of neighboring T4 cells, solitary surviving T4 dendrites established a greater number of likely Mi9-to-T4 connections, distributed more widely across the dendritic arbor. Our results suggest that competitive interactions among T4 cells play a role in refining synaptic compartments, inhibiting the formation or maintenance of incorrect synapses. Without such competition, Mi9-to-T4 connections still preferentially localize to the dendrite tips, but the synaptic compartment is less well-defined. Although early ablation resulted in more pronounced morphological changes, we used the late ablation driver line for GluClα analysis, as it was easier to obtain solitary dendrites suitable for reliable puncta counting. Therefore, for the quantitative analysis, we focused on the dendrites from the late ablation condition. While we did observe a few well-labeled solitary surviving dendrites with the early ablation line (Fig. 4E), these were not included in the quantitative analysis due to their limited number.

### T4 neuron function does not depend on homotypic interactions

**Fig. 5.**
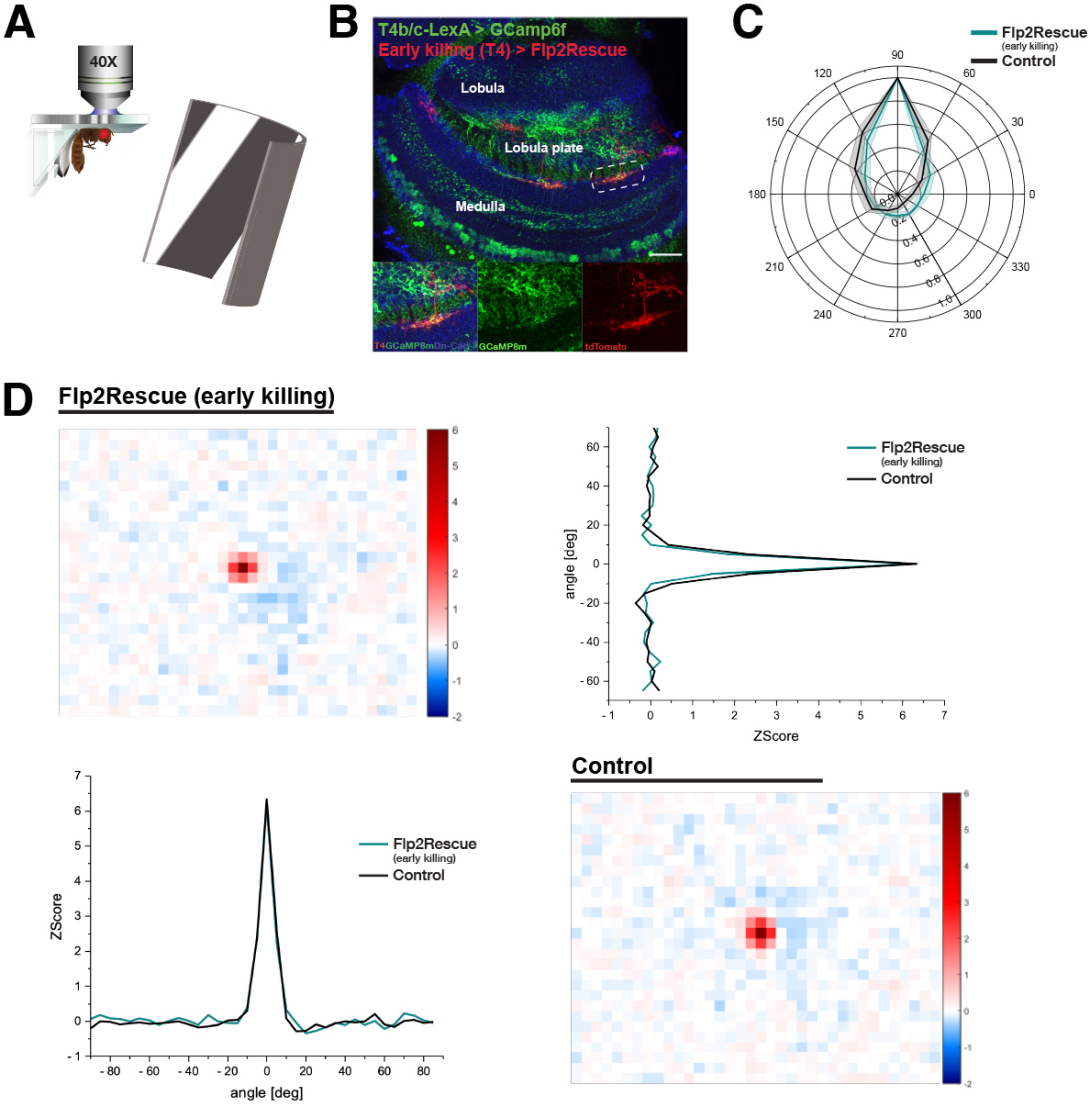
**Directional tuning and receptive field properties of solitary surviving T4 neurons.** (A) Experimental setup for the two-photon calcium imaging in solitary surviving T4 cells. (B) Confocal image of the optic lobe with T4/T5s expressing GcaMP6f (green) and solitary surviving dendrites labeled with tdTomato (red). The brain was recovered after calcium imaging was performed to confirm the solitarity of the dendrites. Insets show higher magnification images of the boxed area. Scale bar: 20 µm. (C) Polar plot of the directional tuning of T4c calcium responses of control (black; flies *n*=5, ROI=18) and early killing solitary surviving dendrites (cyan; flies *n*=8, ROI=17) in medulla. (D) Spatial receptive fields of T4 dendrites in the medulla. Heatmaps depict spatiotemporal receptive fields measured with a white noise flicker stimulus along the azimuth. Line plots on the left and right represent cross-sections along the temporal (horizontal) and spatial (vertical) domain, respectively.

T4 neurons are responsible for detecting motion of light increments, with each subtype tuned to one of the four cardinal directions. To investigate whether the loss of homotypic interactions and the resulting morphological alterations affect T4 neuron function, we measured calcium responses in solitary surviving T4 neurons driving GCaMP expression with the early killing driver line to achieve the strong morphological changes. Isolating signals from a single subtype was challenging because of the spatial proximity of dendrites from different T4 subtypes in the stimulated region,. During confocal imaging, we selected dendrites from the upper medulla region where they appeared solitary; however deeper in the neuropil, we sometimes encountered two or three overlapping dendrites and refrained from imaging them to ensure clean morphological interpretation. During calcium imaging, however, this level of spatial resolution was harder to maintain, as dendritic overlap could not always be excluded with the same precision. To overcome this, we expressed the calcium indicator GCaMP6f specifically in T4b and T4c neurons using a LexA driver. This allowed us to restrict calcium imaging to T4b/c neurons and avoid confounding signals from overlapping dendrites of different T4 subtypes. (The line also targets T5 cells but for simplicity and for our focus we only discuss T4 cells.) Flies were then stimulated with moving gratings, while calcium responses were monitored in T4c dendrites (Fig. 5A). In parallel, some brains were recovered after calcium imaging and stained for confocal microscopy to confirm that the imaged dendrites were indeed solitary (Fig. 5B). To measure receptive fields, we used flies expressing the slower calcium indicator GCaMP8m under the control of the early killing driver, which also labels the solitary surviving T4 neurons.

Although solitary T4 neurons showed clear morphological changes, such as around four times larger dendritic fields, more than doubled elongation along both the A-P and D-V axes and occasional mistargeting into deeper medulla layers, these structural differences did not change the calcium responses of T4b/c neurons. Given the common assumption that dendritic structure and function are closely linked, such expansion might be expected to alter the balance or specificity of inputs. Unexpectedly, we found no significant changes in directional tuning or receptive field properties (Fig. 5C,D). These results suggest that T4 neurons retain their direction-selective function even when their dendritic architecture is altered, showing the robustness of the motion detection circuit. While homotypic interactions seem to refine T4 dendritic growth and targeting, they are not strictly required for establishing direction selectivity.

## DISCUSSION

Our study has shown conclusively that direction-selective T4 neurons regulate the size of their dendritic fields through interactions with their homotypic neighbors. These interactions are particularly crucial for restricting the dendrite size in the early stages of dendrite development, between P30 and P70. While homotypic interactions influence dendritic size, they do not determine the primary subtype-specific orientation of dendrites. Furthermore, we observed an increase in GluClα puncta in solitary surviving dendrites, with a broader distribution along the dendritic arbor rather than being confined to the dendrite tips. Despite these structural changes, the functional properties of these dendrites remained unaffected.

### A new tool for targeted cell ablation in dense populations

T4 neurons are among the most prominent cell types in *Drosophila*, with approximately 3000 neurons per optic lobe. Their subtype-specific morphology and columnar arrangement make them a powerful model for investigating the roles of homotypic interactions. While cell ablation has long been a valuable approach for studying the role of cell–cell interactions in shaping neuronal morphology and circuit assembly (Grueber et al., 2003; Sulston and White, 1980; Gray et al., 2005), many methods such as mechanical laser ablation often lack the precision and reproducibility necessary for reliably targeting dense populations such as T4 cells. There are genetic approaches allowing sparse labeling or targeted ablation of neurons in *Drosophila* (Hampel et al., 2011; Nern et al., 2015); however, they often require co-expression of multiple transgenes (e.g. UAS-hid and a reporter). In contrast, Flp2Rescue integrates both ablation and labeling into a single construct. Genetic approaches such as Gal4/Gal80 (Lee and Luo, 1999; McGuire et al., 2003) can also be leaky or result in incomplete repression. Gal80 suppresses Gal4 activity at the protein level, but its effectiveness depends on precise temporal and expression-level coordination and the repression ability of Gal80 may decline with age (Barwell et al., 2023). Flp2Rescue overcomes these limitations by combining ablation and labeling in a single construct, enabling irreversible, temporally controllable, and reproducible targeting of individual neurons. This makes it a powerful tool for dissecting the role of individual cells especially in small and densely packed neural populations.

### Homotypic interactions constrain dendrite growth

When neighboring T4 dendrites were ablated during development, solitary surviving T4 dendrites expanded their arbors to cover a larger territory. Although T4 dendrites do not exhibit classical tiling and their arbors naturally overlap, this observation suggests that a form of homotypic repulsion may still act to constrain their dendritic growth. The pronounced territory expansion observed following early ablation, compared to late ablation, implies that such interactions present throughout dendrite growth in T4 cells between P30 and P70 (accounting for both the timing of ablation and the time required for clearance of dendrite remnants) (Fig. 3A,B, Figs S2C, S3C). Previous studies have demonstrated that repulsive interactions between branches ensure that dendrites stop growing at an appropriate size (Grueber et al., 2003; Amthor and Oyster, 1995; Sagasti et al., 2005). Thus, the removal of neighboring cells may eliminate an inhibitory cue for dendrite growth. Similar to what has been observed in retinal ganglion cells, for which dendritic size inversely correlates with cell density and for which ablation of neighboring cells leads to dendritic invasion into vacated territory (Perry and Linden, 1982), T4 neurons may rely on those inhibitory local cues from homotypic neighbors to restrict their arbor size.

Interestingly, even as their dendritic fields expanded, T4 dendrites retained their preferred orientation, suggesting that homotypic interactions are unlikely to influence the initial establishment or maintenance of dendritic polarity. This indicates that dendritic orientation is likely governed by intrinsic mechanisms or shaped by heterotypic interactions, such as those between T4 neurons and their presynaptic partners. Or, with the caveat of homotypic neighbors existing before the dendrite growth starts (before P30), interactions could still be important at an earlier stage, but this was beyond the scope of this study. T4 cell fate is specified in progenitors through spatial patterning and Notch-dependent fate decisions, such that newborn neurons already carry their subtype identity. In particular, optomotor-blind (Omb; also known as Bifid) is expressed in distal inner proliferation center neuroblasts and ganglion mother cells that give rise to T4/(5) c,d neurons, reflecting spatial patterning in the proximal inner proliferation center neuroepithelium and is required for establishing c/d identity (Apitz and Salecker, 2016; Pinto-Teixeira et al., 2018). However, it remains largely unclear how these early programs are translated into the morphologically and functionally distinct, subtype-specific features of mature T4 neurons.

The expansion of solitary T4 dendrites may also not solely reflect the loss of homotypic constraints but also a response to increased access to presynaptic inputs. All T4 subtypes receive input from the same set of unicolumnar input neurons in a distinct spatial order (Shinomiya et al., 2019; Borst, 2018; Haag et al., 2016). In the absence of competition from neighboring dendrites, solitary T4 neurons have access to more of these input sites, potentially contributing to both dendritic expansion and the increased number of GluClα puncta we observed. A similar compensatory mechanism has been described in the *Drosophila* giant fiber circuit, where ablation of one class of visual projection neurons leads to dendritic territory expansion and functional compensation by the remaining visual projection neuron types (McFarland et al., 2024). Although this reflects heterotypic, rather than homotypic, plasticity, it shows that developing neurons can adjust their connectivity to maintain circuit function in response to changes in input availability.

### Receptor distribution and functional robustness

Dendrite morphology is thought to influence the organization of synapses and consequently impact neural information processing within a circuit (Lefebvre et al., 2015). In solitary surviving T4 dendrites, we also observed an increased number of GluClα puncta with a broader spatial distribution. This raises the question of how such morphological and synaptic alterations affect dendritic function. Surprisingly, despite the expansion of dendritic arbors and the altered receptor number and distribution, both the receptive field size and directional tuning of T4 neurons remained unchanged. This suggests that the increased GluClα puncta may not correspond to additional functional synapses. They might instead reflect a passive consequence of arbor expansion, leading to a broader but not necessarily active receptor distribution. Without evidence of colocalization with presynaptic markers such as Bruchpilot, the functional status of these receptor puncta remains uncertain.

One possibility is that homotypic interactions normally help define synapse territories and their removal allows receptor spread into previously constrained regions. However, the absence of functional change despite receptor re-distribution suggests that synaptic pruning or stabilization may also rely on activity-dependent mechanisms. Alternatively, the increased GluClα puncta could reflect a form of homeostatic plasticity**,** a compensatory response by the neuron to preserve stable output. Neurons are known to adjust synaptic strength or receptor expression to maintain consistent activity levels under changing input conditions (Turrigiano, 2010; Burrone and Murthy, 2013). In the case of solitary T4 dendrites, the increased number of GluClα puncta may reflect such homeostatic regulation, including compensatory receptor upregulation and formation of additional inhibitory synapses to maintain functional balance. While we specifically imaged GluClα, T4 neurons also express other spatially compartmentalized receptors, such as Rdl and Dα7, which may undergo similar regulation. The re-distribution or upregulation of these receptors may form part of a broader compensatory response to maintain the excitatory–inhibitory balance necessary for directional tuning. Given that T4 directional selectivity depends not only on preferred-direction enhancement but also on null-direction suppression (Haag et al., 2016), preserving the spatial and temporal precision of both excitatory and inhibitory inputs is crucial. Whether through functional synaptic scaling or structural receptor redistribution, these adjustments show the robustness of the T4 circuit in maintaining stable function despite significant morphological change.

### Outlook and future directions

Here, we introduced Flp2Rescue as a general genetic strategy that can be applied to other cell types to study the role of homotypic interactions during neural development. Using this approach, we ablated neighboring T4 neurons at the earliest feasible developmental stage, allowing us to assess the consequences of reduced homotypic neighbors during the main period of T4 dendritic growth. While our results showed several impacts of homotypic neighbors on T4 dendrite development, several questions remain open. In particular, it will be important to determine whether homotypic interactions act broadly among all T4 subtypes or are refined to subtype-specific interactions among T4a-d, as well as to identify the molecular mechanisms mediating these effects. Addressing these questions is a crucial step toward understanding how T4 dendrites develop.

## MATERIALS AND METHODS

### Fly strains

Flies were raised at 25°C and 60% humidity on standard cornmeal agar medium under a 12-h light/12-h dark cycle. At larval and pupal stages, female and male brains were used whereas at adult stages only 1- to 5-day-old female brains were used. Heat shocks to activate hsFLPG5.PEST were applied to young pupas in a 37°C water bath for 5 min (for FRT.CD4-tdGFP activation) and for 13-15 min (to flip the Flp2Rescue construct) or 18 min (for FRT.stopGAL80 inactivation).

The following fly strains were used: tubP.Frt.Gal80 [Bloomington *Drosophila* Stock Center (BDSC) 62103], R42F06-LexA (BDSC 54203), LexAop-rCD2::RFP, UAS-mCD8::GFP (BDSC 67093), VT16255-Gal4.AD (BDSC 75205), VT12314-Gal4.DBD (BDSC 74478), R39H12-Gal4.DBD (BDSC 69444), VT043070-Gal4.AD (BDSC 71655), R42F06-Gal4.DBD (BDSC 69285), hsFLPG5.PEST (BDSC 62118), UAS-GCaMP6f (BDSC 42747), UAS-GCaMP8m (BDSC 92590), UAS-GluClα::GFP in attP1 (Fendl et al., 2020), UAS-hid in attP1 (this study), FRT.stop-UAS-CD4-tdTomato in attP1 (this study), Flp2Rescue in su(Hw)attP5 (this study), LexAop-myr-tdTomato in su(Hw)attP5 (this study), LexAop-GCaMP6f (44277), UAS-CD4-tdTomato (BDSC 35837), UAS-myr- tdTomato (BDSC 32222), GMR71D01-LexA (Tm3) (BDSC 52709), GMR64B07-LexA (Mi1) (BDSC 39293).

### Crosses

For input cell layer targeting, Tm3-LexA LexAop-RFP UAS-GFP; UAS-Flp2Rescue was crossed to VT16255-AD; VT12314-DBD, and Mi1-LexA LexAop-RFP UAS-GFP; UAS-Flp2Rescue was crossed to VT16255-AD; VT12314-DBD. For tracking T4 cell remnants during development, R42F06-LexA LexAop-rCD2::RFP UAS-GFP; UAS-Flp2Rescue was crossed to VT16255-AD; VT12314-DBD (late killing) and VT16255-AD; R39H12-DBD (early killing) separately. For solitary surviving dendrites with early killing, hsFLPG5.PEST; VT16255-AD; VT12314-DBD was crossed to w; UAS-Flp2Rescue (+heat shock). For solitary surviving dendrites with late killing, hsFLPG5.PEST; VT16255-AD; R39H12-DBD was crossed to w; UAS-Flp2Rescue (+heat shock). For GluClα analysis of solitary surviving dendrites, w; UAS-Flp2Rescue; UAS-GluClα::GFP was crossed to late killing Gal4. For GluClα single control dendrites, hsFLPG5.PEST; VT16255-Gal4; UAS-FRT.stop-Gal80 was crossed to w; UAS-GluClα::GFP; UAS-tdTomato (+ heat shock). For GluClα control with GluClα distribution, hsFLPG5.PEST; VT16255-Gal4 was crossed to w; UAS-GluClα::GFP; UAS-FRT.stop-tdTomato. For control T4 dendrites, hsFLPG5.PEST; VT16255-AD; R39H12-DBD was crossed to w; UAS-FRT.stop-tdTomato. For two-photon imaging, w; UAS-Flp2Rescue; T4T5c-LexA, LexAop-GCaMP6f was crossed to early killing Gal4. For receptive field measurements, w; UAS-Flp2Rescue; UAS-GCaMP8m was crossed to early killing Gal4. T4/T5-LexA was crossed to R42F06-LexA; LexAop-rCD2::RFP.

### Generation of Flp2Rescue construct

The Flp2Rescue construct was generated from the backbone of the pJFRC7-20XUAS-IVS-mCD8::GFP plasmid (Addgene plasmid #26220), which was digested with NotI and XbaI. GENEWIZ generated two DNA fragments, which were inserted into the digested backbone. Fragment one contained half of the flex-switch FRT FRT14 followed by hid. Fragment two contained an inverted CD4-tdTomato followed by FRT14 FRT. Embryo injections were performed by BestGene Inc., including PCR-verifications and balancing.

### Antibodies and immunolabeling

Primary antibodies used in this study were: alpaca GFP-Booster ATTO647N (1:300; ChromoTek, gba647n), chicken anti-GFP (1:600; Thermo Fisher Scientific, A10262), rat anti-DN-Cadherin (1:50; Developmental Studies Hybridoma Bank, DN-Ex#8), mouse anti-Connectin (1:50; Developmental Studies Hybridoma Bank, C1.427) and rabbit anti- DsRed (1:1000; Clontech, 632496). Secondary antibodies used in this study (all used 1:500) were: Alexa Fluor 488-conjugated goat anti-mouse (Thermo Fisher Scientific, A28175), Alexa Fluor 488-conjugated goat anti-rat (Invitrogen, A11006), Alexa Fluor 488-conjugated donkey anti-chicken (Jackson ImmunoResearch, 703-545-155), Alexa Fluor 568-conjugated goat anti-rabbit (Life Technologies, A11011), Alexa Fluor 568-conjugated goat anti-mouse (Invitrogen, A11004), ATTO 647N-conjugated goat anti-mouse (Rockland Immunochemicals, 610-156-040) and Alexa Fluor 647-conjugated goat anti-rat (Life Technologies, A21247).

### Immunohistochemistry

For immunolabeling, brains were dissected in cold PBS supplemented with 0.3% Triton X-100 (PBST) within a time limit of 30 min. Subsequently, the brains were fixed with 4% paraformaldehyde for precisely 30 min at room temperature and then rinsed three times for 15 min each with PBST. Before applying the respective antibodies, the brains were blocked in PBST containing 10% normal goat serum (Sigma-Aldrich) or 10% fetal bovine serum (Thermo Fisher Scientific) for at least 30 min at room temperature. A 200 μl primary antibody solution was then added to the brains, which were then incubated for approximately 24-48 h at 4°C. Following incubation, the brains were thoroughly washed several times for 3 h each, and a 200 μl secondary antibody solution was applied. The brains were once again incubated for approximately 24-48 h at 4°C and subjected to several rounds of washing with PBT for 3 h each. Brains then were washed once with PBS, before being mounted in Gold Antifade Mountant (Invitrogen, S36937) with 22×22 mm glass coverslips. Whole brains were mounted without sectioning under No. 1.5 coverslips.

### Confocal imaging, image processing and visualization

Confocal imaging was performed using a Leica Stellaris SP8 laser scanning confocal microscope using the 488 nm, 561 nm and 633 nm lasers and a 63× glycerol-immersion objective (Leica, 11506353). Some dendrites were imaged with Leica Lightning deconvolution to achieve higher resolution. The images were captured with an *xy* resolution of approximately 69 nm and a *z* step of 500 nm at a resolution of 1024×1024 pixels. Image processing and quantitative analyses were performed with the Fiji software package.

Quantification of GFP signal during development was done by analyzing confocal images in Fiji. For each optic lobe, the same anatomical region was quantified across conditions by selecting a region of interest (ROI) (same structure and comparable *z*-range across samples). Mean fluorescence intensity was measured for the GFP channel and for Bruchplot (nc82) within the ROI using Fiji*.* To account for sample variability in staining and imaging, a per-optic lobe normalization was performed by computing the ratio GFP/nc82 for each brain. These values were then normalized to the mean adult control value to generate relative GFP intensity (adult control set to 1.0), enabling comparison across time points and conditions.

### Two-photon microscopy

Fly microsurgeries were carried out on female flies, and functional calcium imaging of T4 neurons in the right optic lobe was performed as described by Maisak et al. (2013). Flies were cold-anesthetized and mounted on a Plexiglas holder using beeswax, with the thorax and legs immobilized. The head was gently bent downward to expose the posterior part and inserted into a custom-cut opening in aluminum foil, which was clamped into a recording chamber. The chamber was filled with external saline, and a small window was cut on the left side of the head capsule. Muscles, adipose tissue, and trachea were removed to expose the optic lobe. Two-photon images were acquired using ScanImage 3.8 (Vidrio Technologies) running in MATLAB R2013b (MathWorks).

### Direction tuning

We used a custom-built visual stimulation arena based on a projection system with two mirrors and a bowl-shaped screen, following the design described by Arenz et al. (2017). Visual stimuli were projected onto the back of an opaque cylindrical screen using two commercial micro-projectors (TI DLP Lightcrafter 3000), which provided flexible stimulus presentation across 180° in azimuth and 105° in elevation of the visual field of the fly. The projectors were operated using only the green LED (500-600 nm), and two long-pass filters (Thorlabs FEL0550 and FGL550) were placed in front of a projector to restrict stimulus light to wavelengths above 550 nm. Calcium imaging was performed using GCaMP6f and GcaMP8m. To isolate the GCaMP emission signal, a band-pass filter (Brightline 520/35) was placed in front of the photomultiplier. Additional black cardboard shielding was used to block stray light from the arena to suppress any leak of the arena light into the photomultiplier signal. The maximum luminance achieved was 276±48 cd/m².

For measuring the direction tuning, square wave gratings with a wavelength of 30 deg were shown to flies in 12 directions at a velocity of 15 deg/s. Calcium signals were recorded from dendrites of T4c neurons with a 64×64-pixel resolution at a frame rate of 13 Hz.

Data were analyzed using custom-written routines in MATLAB (R2023b). Motion correction and calculation of ΔF/F in ROIs were performed using previously described methods (Arenz et al., 2017). The ΔF/F values of each ROI were averaged over three stimulus repetitions. To quantify the extent of directional tuning for each ROI, the directional tuning index (L_dir_) was calculated from vectors representing peak responses, as the magnitude of the resultant vector divided by the sum of the individual vectors' magnitudes using the following equation:

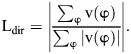
L_dir_ can range from 0 to 1, corresponding to absence of direction selectivity and maximum direction selectivity, respectively.

### Receptive fields

Compared to the previously described system for visual stimulation (Prech et al., 2024), we utilize a nearly distortion-free method for measuring receptive fields. For this purpose, a projector (LG PH510PG) in combination with two mirrors (ME8S-G01–8″, Thorlabs) was used to create an inside-out projection of the visual stimulus onto a custom-built screen. This specific arrangement and geometry allow for the virtual presentation of a spherical texture to the fly, embedding them within a virtual reality. To merge two-photon microscopy and bowl-based visual stimulation, the blue and red LEDs of the projector were disabled with custom written software. Aligned with the previous configuration, the projected light was spectrally filtered using the same optical filters (Thorlabs FEL0550 and FGL550). In addition, the original setup was expanded by incorporating a cardboard housing, ensuring effective shielding against stray light. Luminance and geometric accuracy were measured at the position of the fly using a 360° camera, as described in Prech et al. (2024).

For receptive field mapping, we used GCaMP8m with a resolution of 32×32 pixels and a frame rate of 25 Hz for neural recording. Responses of T4c neurons were measured while presenting binary white noise stimuli mapped onto a spherical texture over a duration of 5 min. The white noise stimuli, with a spatial resolution of 5° solid angle, segmented the field of view of the arena into 36×28 pixel. Pixel intensities were randomly assigned to either 0% or 100% luminance and updated every 40 ms (25 Hz). By applying a sliding reverse correlation after 64×64 pixel resolution acquisition, we reconstructed the linear contribution of each pixel to the recorded signal. The resulting receptive field images represent equidistant cylindrical projections of the spatial response profile. To achieve distortion-free alignment of the resulting receptive fields, a correction using inverse rotation was performed as previously described by Prech et al. (2024).

### Pre-processing and image analysis

Pre-processing of images defining a single automated pipeline that works effectively for all images is a challenge, so a semi-automated pipeline was developed entailing a number of steps whereby an expert reviewer could optimize parameters to ensure accurate single dendrite images. First, a mask is defined around each individual dendrite by first spatially smoothing with a Gaussian filter before applying generalized histogram thresholding (Barron, 2020 preprint) in order to binarize the image and extracting the largest connected region of pixels. At this stage, the reviewer can manipulate the width of the Gaussian kernel used for smoothing in order to ensure a masked region is defined that encompasses each single dendrite. Second, the mask is applied to the original unedited image. At this stage, the reviewer can apply contrast enhancement if needed, using CLAHE, before the masked region is again thresholded using generalized histogram thresholding. In some cases, CLAHE was applied again post-thresholding to improve the image contrast. This results in a single dendrite extracted from each image, which can be used for further analysis. In order to determine the two-dimensional area spanned by a single dendrite, a convex hull is calculated for each dendrite. Density is then calculated as the ratio of non-zero pixels within the hull over total pixels within the hull. In order to calculate the elongation along each axis for different dendrites, rotation of the imaging plane has to be accounted for. In order to do this, each image is aligned to its eigenvectors calculated from the covariance matrix of pixel coordinates, to give the intrinsic orientation axis for each dendrite. The extent of pixel spread along each axis is then used to represent dorsal/ventral and anterior/posterior spread of each dendrite, allowing for the comparison of elongation/contraction of the dendrite. Similarly, circularity of each dendrite is calculated as the absolute difference between normalized eigenvalues, where a value of 0 would correspond to a circle and a value of 1 would equate to a straight line. In order to estimate the projection direction of each dendrite, the angle between the start point of the dendrite and each pixel is calculated, relative to the imaging plane. For each dendrite the angular mean and variance is then calculated as described by Amthor and Oyster (1995). For histogram plots, angles are binned into 100 bins between 0° and 360°.

### Synapse analysis

The numbers of glutamatergic receptor puncta were counted as number of GFP puncta overlapping with the tdTomato signal labeling the dendrite branches. The puncta were counted manually using Fiji 3D projection. The distribution of glutamatergic along the proximodistal axis of T4 dendrites was analyzed as described by Fendl et al. (2020). Relative expression levels of receptors were quantified as follows: for each brain, we measured the mean gray values (anti-GFP booster) in medulla M10 normalized to background gray values. The area was drawn around T4 dendrites.

### Statistical analysis and graphs

Calculations were performed and plots were generated with Python 3.9 using the SciPy, Numpy, Matplotlib, Pandas and Seaborn packages. For statistical analysis, we performed a permutation-based two-way ANOVA to test for main effects of experimental condition and T4 neuron subtype, and their interaction. *P*-values were obtained from 5000 permutations (Freedman–Lane residual permutation procedure). To assess condition effects within each subtype, we performed permutation-based pairwise comparisons of the conditions separately within subtypes (A-D). For receptor puncta distribution, we performed Mann–Whitney test. Resulting *P*-values were adjusted for multiple testing across subtypes using the Holm method (family-wise error control). Asterisks indicate significance as follows: *P≤0.05; **P≤0.01; ***P≤ 0.001. Figures were prepared using Adobe Illustrator software.

## Supplementary Material

10.1242/develop.205238_sup1 Supplementary information
