# Supplementary material for: Homotypic dendritic interactions constrain growth and receptor distribution in Drosophila T4 neurons without affecting orientation or function
Source: Development. 2026 Apr 8;153(7):dev205238. doi: 10.1242/dev.205238 (PMC13096777; doi:10.1242/dev.205238)
Supplement: Supplementary information [file develop-153-205238-s1.pdf]

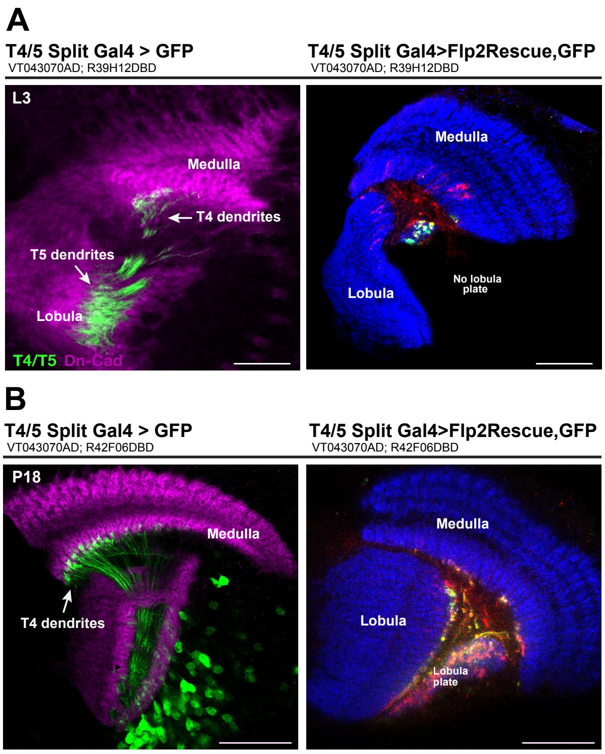

Fig. S1. T4/T5 neurons are required for the proper lobula plate formation

Development • Supplementary information

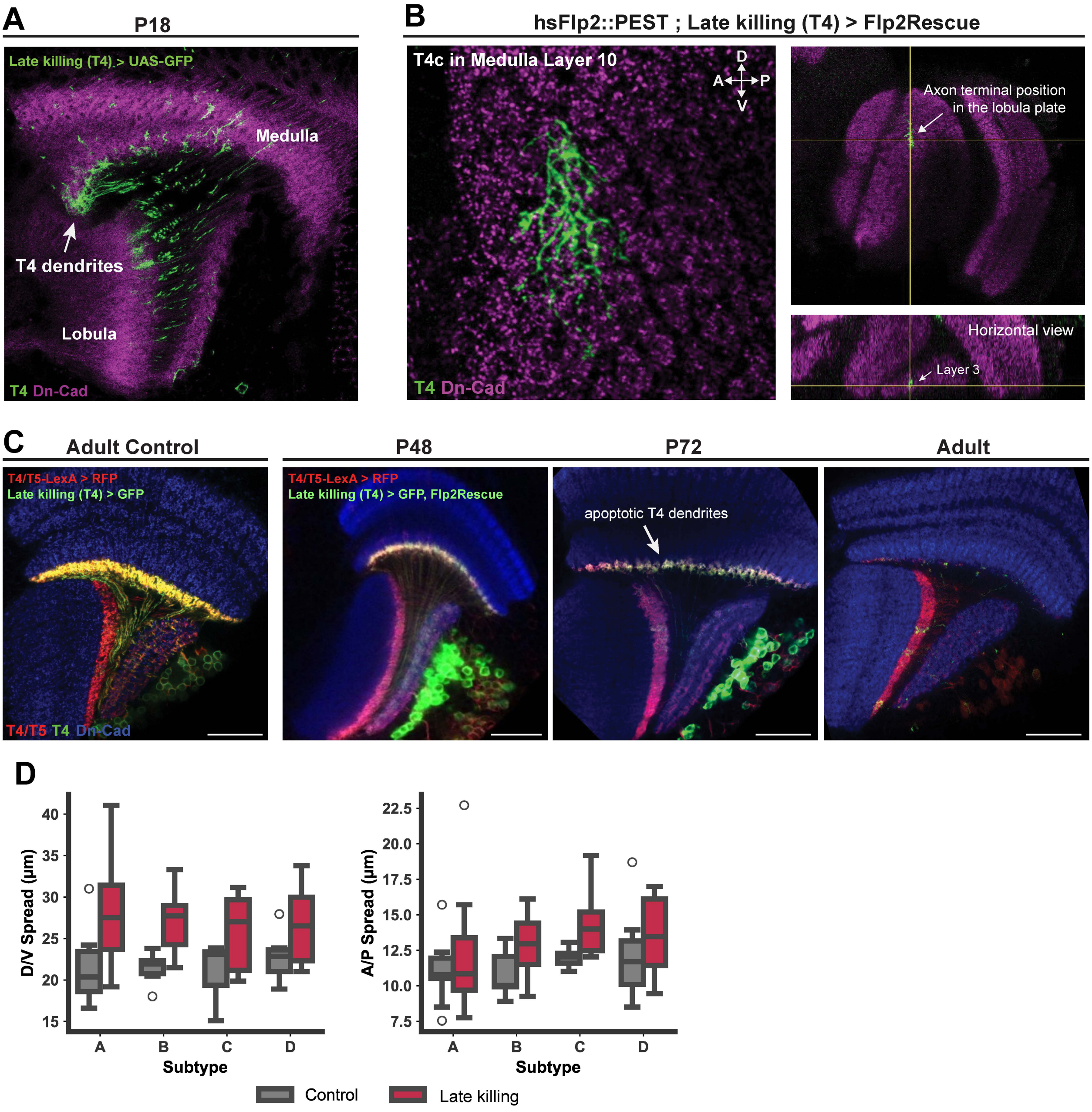

Fig. S2. Onset of the late killing driver line, setup for single T4 dendrite imaging and axon terminal position confirmation and removal of dendrite remnants upon ablation induction

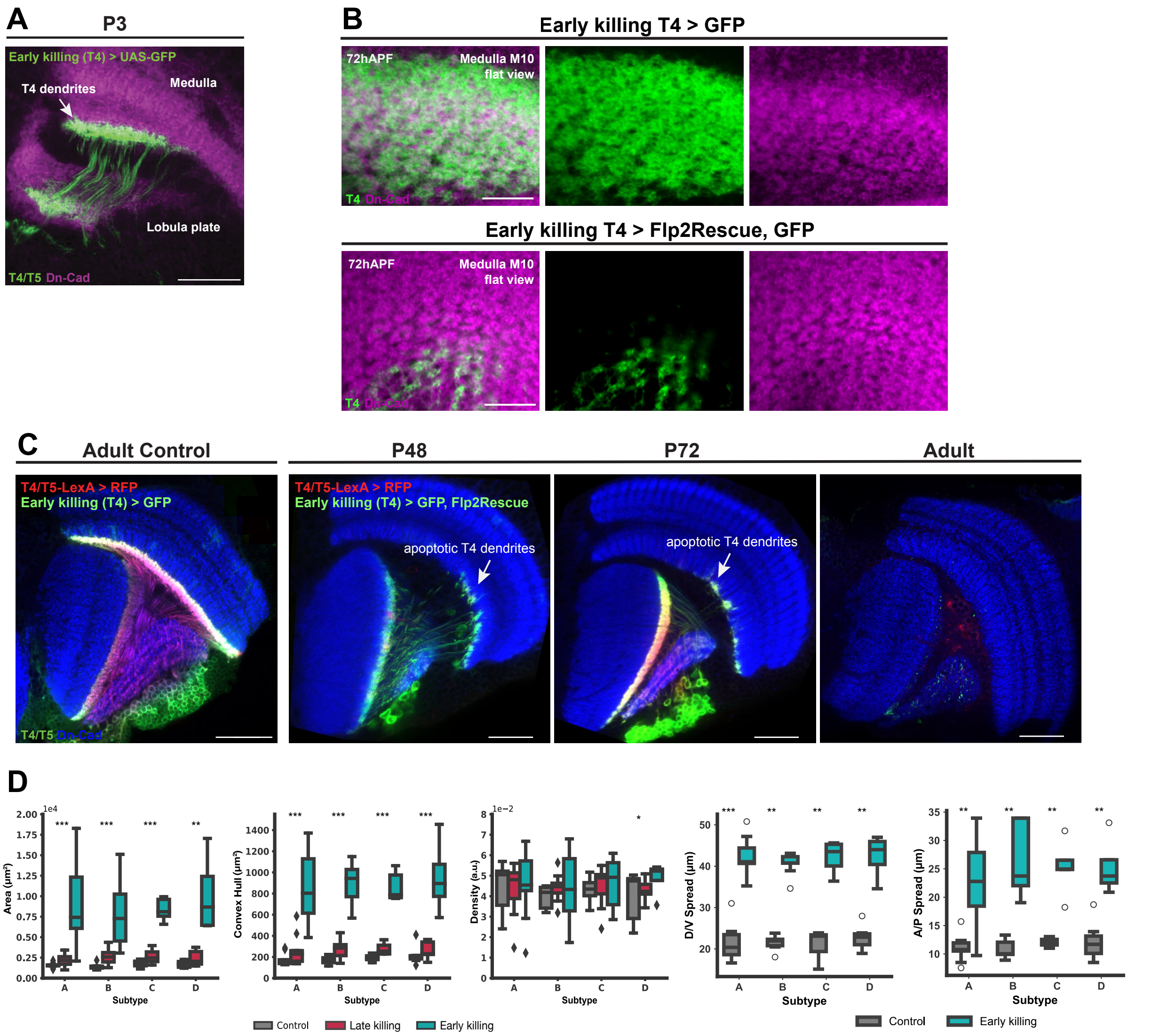

Fig. S3. Onset of the early killing driver line and removel of T4 cell remnants upon ablation induction

Development • Supplementary information
